# Water Typhoid

**Published:** 1901-08-24

**Authors:** 


### WATER TYPHOID.

Speaking at Cheltenham, Dr. Reynolds, Commissioner of Health, Chicago, said that Chicago was supplied with water from Lake Michigan by four tunnels under the lake ranging in length from two to four miles. Each day a sample of water collected from the shore end of each tunnel was carefully analysed both chemically and bacteriologically, and it was found that when any pollution of the water was discovered this was followed by a rise in the mortality from typhoid fever and from acute intestinal diseases, the rise taking place in the case of typhoid fever in about five weeks and in the case of the other diseases in about one week. This seems to be a matter of considerable importance in view of the statement so often made, a statement which has received the endorsement of at least one Royal Commission, that it is inconceivable that the infection of so large a mass of water as the Thames could have any influence upon the typhoid mortality of communities drawing their supply from it. As clinching the matter, Dr. Reynolds pointed out that since the main sewerage of Chicago had been diverted from the lake and made to discharge into the Mississippi Valley the typhoid mortality in the city had been greatly reduced. Until recently the majority of the sewers of the city emptied into the Chicago river and a few directly into the lake. In January 1900 the waterway which had been cut through the divide between the St. Lawrence Valley, of which the great lakes form a part, and the Mississippi Valley was opened, and since then the waters of the Chicago river and of Lake Michigan have flowed away from the city down the Mississippi Valley and carried with them all the city sewage except that emptying directly into the lake. The result has been that the death-rate in the city from typhoid fever has been greatly reduced; all of which goes to prove that much of the previous mortality had been due to sewage pollution of the water-supply, notwithstanding that it was derived from a great lake through culverts opening from two to four miles out. In view of this statement by Dr. Reynolds, and the confirmation which it affords to the opinion that sewage pollution, even when flowing into a very large mass of water, is attended with serious risks to those communities which derive their supply from such a source, and that no amount of dilution which is attainable in practice is capable of removing this risk, it is very satisfactory to note the great efforts which are at present being made to ensure the purity of the river Thames. This is largely due to the persistent action of the Thames Conservancy in compelling towns and villages within the area controlled by them to divert their sewage from the river. During the past year 32 towns and villages, with an aggregate population of more than 80,000, have thus diverted their sewage, and in many other instances pressure has been brought to bear upon local authorities whose arrangements in this regard were unsatisfactory. In only 16 cases was it necessary to institute legal proceedings, the authorities having in most cases undertaken to do what was required of them. Good progress is being made but much still remains to be done, for as old sources of pollution are removed new ones are constantly cropping up. At the very time that village drainage is being diverted on to sewage farms at great expense, a new source of pollution is arising in the fact that during the summer months the whole length of the Thames and its banks is turned into a vast pleasure ground, and when people go a-pleasuring they are none too particular about the niceties of life.

